# Supplementary material for: Mechanisms governing the pioneering and redistribution capabilities of the non-classical pioneer PU.1
Source: Nat Commun. 2020 Jan 21;11:402. doi: 10.1038/s41467-019-13960-2 (PMC6972792; doi:10.1038/s41467-019-13960-2)
Supplement: Supplementary file 7 — Source data [file 41467_2019_13960_MOESM7_ESM.zip › Source_Data/FigureS2/FigureS2B_MotifScanOutput/KmeansCluster2/homerResults/motif1.similar.html]

motif1

## Information for motif1

C
T
G
A
C
G
T
A
C
G
T
A
C
G
T
A
C
T
A
G
T
C
G
A
C
T
A
G
A
C
T
G
C
G
T
A
C
G
T
A
T
A
C
G
G
C
A
T
T
A
C
G
T
G
C
A
  
Reverse Opposite:  

A
C
G
T
A
T
G
C
C
G
T
A
A
T
G
C
C
G
A
T
C
G
A
T
A
G
T
C
A
G
T
C
A
G
C
T
G
A
T
C
G
C
A
T
G
C
A
T
G
C
A
T
G
A
C
T
  

|  |  |
| --- | --- |
| p-value: | 1e-2879 |
| log p-value: | -6.630e+03 |
| Information Content per bp: | 1.627 |
| Number of Target Sequences with motif | 3838.0 |
| Percentage of Target Sequences with motif | 76.41% |
| Number of Background Sequences with motif | 2111.1 |
| Percentage of Background Sequences with motif | 5.19% |
| Average Position of motif in Targets | 102.0 +/- 22.9bp |
| Average Position of motif in Background | 99.8 +/- 75.0bp |
| Strand Bias (log2 ratio + to - strand density) | -0.1 |
| Multiplicity (# of sites on avg that occur together) | 1.08 |
| Motif File: | file (matrix) reverse opposite |

### Similar de novo motifs found

|  |  |  |  |  |  |  |  |
| --- | --- | --- | --- | --- | --- | --- | --- |
| Rank | Match Score | Redundant Motif | P-value | log P-value | % of Targets | % of Background | Motif file |
| 1 | 0.931 | C T G A A T G C G A C T G C A T T G A C G A T C A G C T A G T C G C A T G C A T G C A T | 1e-2564 | -5904.849575 | 94.76% | 19.48% | motif file (matrix) |
| 2 | 0.944 | T C G A C T G A C T G A C G T A T C A G T C G A C T A G A C T G C G T A C G T A T A C G C G A T T A C G | 1e-2562 | -5900.465190 | 79.00% | 8.41% | motif file (matrix) |
| 3 | 0.973 | T A G C G C T A A T G C A C G T A C G T A G T C A G T C A G C T G A T C G C A T G A C T G C A T | 1e-2482 | -5716.237467 | 86.32% | 13.38% | motif file (matrix) |
| 4 | 0.932 | C T G A C G T A T C A G T C G A C T A G A C T G C G T A C T G A T A C G C G A T T A C G | 1e-2345 | -5400.929759 | 78.32% | 9.82% | motif file (matrix) |
| 5 | 0.922 | A T G C G C T A A T G C A C G T A C G T A G T C A G T C G A C T A G T C G C A T | 1e-2344 | -5398.361845 | 81.07% | 11.38% | motif file (matrix) |
| 6 | 0.920 | A T G C C G T A A T G C A C G T A C G T A G T C A G T C A G C T A G T C | 1e-2330 | -5366.945069 | 83.20% | 12.82% | motif file (matrix) |
| 7 | 0.928 | C T G A C T G A T C A G T C G A C T A G C T A G C G T A T C G A T A C G C G A T T A C G T C A G | 1e-2106 | -4849.281232 | 72.17% | 8.73% | motif file (matrix) |
| 8 | 0.885 | C G T A A T G C A G C T C G A T G T A C G A T C A G T C A G T C G C A T G C A T | 1e-2058 | -4739.169065 | 97.41% | 30.18% | motif file (matrix) |
| 9 | 0.879 | T C A G C T G A A C T G A C T G C G T A C G T A A T C G G C A T | 1e-1859 | -4281.668098 | 86.94% | 21.47% | motif file (matrix) |
| 10 | 0.903 | A T G C C G T A A G T C A C G T A C G T A G T C A G T C | 1e-1681 | -3872.076954 | 63.75% | 8.45% | motif file (matrix) |
| 11 | 0.921 | A T G C G T C A A G T C A C G T A C G T A G T C A G T C G A C T | 1e-1499 | -3451.697986 | 65.70% | 11.34% | motif file (matrix) |
| 12 | 0.877 | C T G A A C T G A C T G C G T A C G T A A T C G A G C T | 1e-1480 | -3408.021031 | 81.49% | 22.60% | motif file (matrix) |
| 13 | 0.897 | T C G A A T G C A C G T A C G T A G T C A G T C G A T C G A T C G T C A | 1e-1470 | -3386.122555 | 87.42% | 28.41% | motif file (matrix) |
| 14 | 0.844 | T C A G T A C G C T A G C T A G C G T A C G T A C T A G A C G T | 1e-1468 | -3380.940981 | 68.25% | 13.19% | motif file (matrix) |
| 15 | 0.869 | C T G A C G T A T A C G C G T A C T A G A T C G T G C A C T G A T A C G A G C T G C A T T C A G | 1e-1262 | -2905.947595 | 68.94% | 16.50% | motif file (matrix) |
| 16 | 0.776 | A C G T A C G T A G T C A G T C A G T C A T G C G A C T A C G T A G C T | 1e-1121 | -2581.458780 | 61.80% | 13.98% | motif file (matrix) |
| 17 | 0.813 | G C A T C A G T A T G C A G T C G A C T A G T C C G A T C G A T G A C T A G C T | 1e-1120 | -2580.052462 | 52.04% | 8.61% | motif file (matrix) |
| 18 | 0.774 | C G A T A C G T T A G C A G T C A G C T A G C T C G A T C G A T | 1e-1017 | -2343.448487 | 66.89% | 19.13% | motif file (matrix) |
| 19 | 0.849 | T C G A T G C A C G T A C T A G C T A G T A C G T G C A C G T A T A C G G C A T C A T G C T G A A C G T | 1e-880 | -2027.720768 | 55.01% | 13.55% | motif file (matrix) |
| 20 | 0.823 | G C T A C A G T T A C G T C A G A C T G G C T A G C T A T A C G G A C T | 1e-863 | -1987.840002 | 38.32% | 5.27% | motif file (matrix) |
| 21 | 0.785 | A C T G C T G A C T A G A C T G C G T A C T G A A T C G | 1e-848 | -1952.605733 | 63.37% | 19.80% | motif file (matrix) |
| 22 | 0.820 | C A T G T C G A C T A G A C T G C G T A C G T A T A C G A T G C | 1e-719 | -1657.128356 | 52.60% | 14.88% | motif file (matrix) |
| 23 | 0.928 | C G A T A C G T C G A T C T A G C T G A A C T G A C T G C G T A C G T A T A C G C G A T T A C G T G A C | 1e-643 | -1481.235991 | 27.91% | 3.41% | motif file (matrix) |
| 24 | 0.820 | T A C G C T G A C A T G A C T G G T C A C G T A T A G C C G A T A C T G T A G C G T A C | 1e-515 | -1185.932679 | 23.49% | 3.03% | motif file (matrix) |
| 25 | 0.769 | C T A G C A T G A G T C A C G T A C G T A G T C A G T C A C G T A T G C C G T A A C G T A G C T G C A T A T G C | 1e-468 | -1079.317694 | 23.81% | 3.64% | motif file (matrix) |
| 26 | 0.842 | A C T G C G T A C T G A C T A G A C T G C G T A C G T A A T C G C G A T A T C G | 1e-376 | -866.144848 | 24.09% | 4.98% | motif file (matrix) |
| 27 | 0.782 | C A G T A C T G C G T A C T G A T C A G A C G T T A C G C G T A C G A T | 1e-374 | -862.083525 | 28.53% | 7.24% | motif file (matrix) |
| 28 | 0.848 | C G T A T C G A G T C A G C T A C T G A C T A G T A C G C T G A C G T A C A T G A C G T T C A G C T G A | 1e-177 | -408.837925 | 8.40% | 1.05% | motif file (matrix) |
| 29 | 0.759 | C T G A C T G A T G C A C G T A C T A G A T G C C G T A A C T G C G T A C G T A T A C G C G A T T A C G | 1e-165 | -380.027530 | 6.31% | 0.52% | motif file (matrix) |
| 30 | 0.758 | A G C T T A G C G T C A A T G C G A C T C A G T G T A C G A T C G T A C G C A T A G T C | 1e-151 | -349.668296 | 8.90% | 1.53% | motif file (matrix) |
| 31 | 0.828 | A G T C C T G A A C T G A C G T A C G T A G T C A G T C | 1e-149 | -343.807422 | 25.92% | 11.53% | motif file (matrix) |
